# Farmers' preferences for high-input agriculture supported by site-specific extension services: Evidence from a choice experiment in Nigeria

**DOI:** 10.1016/j.agsy.2019.02.003

**Published:** 2019-07

**Authors:** Oyakhilomen Oyinbo, Jordan Chamberlin, Bernard Vanlauwe, Liesbet Vranken, Yaya Alpha Kamara, Peter Craufurd, Miet Maertens

**Affiliations:** a Division of Bio-economics, Department of Earth and Environmental Sciences, KU Leuven, Celestijnenlaan 200E-box 2411, 3001 Heverlee, Belgium; b International Maize and Wheat Improvement Center (CIMMYT), P.O. Box 5689, Addis Ababa, Ethiopia; c International Institute of Tropical Agriculture (IITA), P.O. Box 30772-00100, Nairobi, Kenya; d International Institute of Tropical Agriculture (IITA), P.M.B. 3112, Kano, Nigeria; e International Maize and Wheat Improvement Center (CIMMYT), P.O. Box 1041-00621, Nairobi, Kenya

**Keywords:** Agricultural technology adoption, Agricultural extension, ICT-based extension, Site-specific extension, Soil fertility management, Maize yield

## Abstract

Agricultural extension to improve yields of staple food crops and close the yield gap in Sub-Saharan Africa often entails general recommendations on soil fertility management that are distributed to farmers in a large growing area. Site-specific extension recommendations that are better tailored to the needs of individual farmers and fields, and enabled by digital technologies, could potentially bring about yield and productivity improvements. In this paper, we analyze farmers' preferences for high-input maize production supported by site-specific nutrient management recommendations provided by an ICT-based extension tool that is being developed for extension services in the maize belt of Nigeria. We use a choice experiment to provide ex-ante insights on the adoption potentials of site-specific extension services from the perspective of farmers. We control for attribute non-attendance and account for class as well as scale heterogeneity in preferences using different models, and find robust results. We find that farmers have strong preferences to switch from general to ICT-enabled site-specific soil fertility management recommendations which lend credence to the inclusion of digital technologies in agricultural extension. We find heterogeneity in preferences that is correlated with farmers' resource endowments and access to services. A first group of farmers are strong potential adopters; they are better-off, less sensitive to risk, and are more willing to invest in a high-input maize production system. A second group of farmers are weak potential adopters; they have lower incomes and fewer productive assets, are more sensitive to yield variability, and prefer less capital and labor intensive production techniques. Our empirical findings imply that improving the design of extension tools to enable provision of information on the riskiness of expected outcomes and flexibility in switching between low-risk and high-risk recommendations will help farmers to make better informed decisions, and thereby improve the uptake of extension advice and the efficiency of extension programs.

## Highlights

• Choice experiement on farmer preferences for site-specific extension recommendations
• High willingness to accept ICT-enabled and site-specific extension recommendations
• Observed heterogeneity in farmers' willingness to intensify production systems
• Information on riskiness of return crucial for improved design of extension tools

## 1 Introduction

The yields of major food crops, such as maize, in Sub-Saharan Africa (SSA) are lagging behind yields in other parts of the world, and are often far below their potential (Tittonell and Giller, 2013; Vanlauwe et al., 2015a; Guilpart et al., 2017). This contributes to persistent poverty among smallholder farmers, slow agricultural growth and dependency on food imports, and food insecurity among a rapidly growing population (Barrett and Bevis, 2015; van Ittersum et al., 2016; Komarek et al., 2017; Ragasa and Mazunda, 2018). Poor soil fertility is a major biophysical factor limiting maize yields in SSA in general (Kihara et al., 2016) and in Nigeria in particular (Shehu et al., 2018). Nutrient-related constraints in maize production include macronutrient (nitrogen (N), phosphorus (P) and potassium (K)) deficiencies, especially N, as well as secondary nutrient and micronutrient deficiencies and soil acidity (Kihara et al., 2016; Vanlauwe et al., 2015b).

Improving soil fertility is challenging because of the large spatio-temporal heterogeneity in biophysical and socio-economic conditions of smallholder farming systems (Tittonell et al., 2010; Vanlauwe et al., 2015b; Njoroge et al., 2017; MacCarthy et al., 2018). Given an average low level of input use, it is often argued that smallholder farmers in SSA need to intensify the use of external inputs, especially inorganic fertilizer, in order to improve yields and productivity (Chianu and Tsujii, 2005; Duflo et al., 2011; Wiredu et al., 2015; Sheahan and Barrett, 2017). Yet, empirical findings for Nigeria (Liverpool-Tasie et al., 2017), Kenya (Sheahan et al., 2013) and Zambia (Burke et al., 2017) show that this argument does not always hold and that it is not always profitable for farmers to increase their application rates of inorganic fertilizer in maize production, primarily because of a low maize yield response to fertilizer application in some areas. These studies argue that a low marginal physical product of applied N is a more important factor limiting the profitability and the use of fertilizer in some regions than market-related and institutional constraints such as high transaction costs, and imperfections in credit and input markets. Extension services on soil fertility management that are adapted to the local context of individual farmers may contribute to improving the yield response to fertilizer and the marginal physical product of applied fertilizer (Vanlauwe et al., 2015b).

Yet, in SSA, and elsewhere, agricultural extension most often entails general recommendations for improved soil fertility management that are disseminated to farmers in a large growing area, covering e.g. a region, a district or a province (Tittonell and Giller, 2013; Kihara et al., 2016; Shehu et al., 2018). Such agricultural extension practices fail to take into account the heterogeneous and complex biophysical and socio-economic conditions of smallholder farming (MacCarthy et al., 2018; Kihara et al., 2016). Site-specific agricultural extension, on the other hand, includes recommendations that are tailored to the situation of an individual farmer or field. Such recommendations might be more effective to bring about yield and productivity improvements than conventional extension practices (Ragasa and Mazunda, 2018). To improve the capacity of agricultural extension providers in the delivery of site-specific extension recommendations to farmers, information and communication technology (ICT) driven decision support tools (DSTs) offer great potential (Kragt and Llewellyn, 2014; Vanlauwe et al., 2015b; Vanlauwe et al., 2017). The role of digital technologies in agriculture in developing countries is increasing (Bernet et al., 2001; Fu and Akter, 2016; Verma and Sinha, 2018) and these technologies might provide a cost-effective and innovative way to providing site-specific fertilizer recommendations to smallholder farmers (Njoroge et al., 2017).

In this paper, we analyze farmers' preferences for high-input production systems supported by site-specific nutrient management (SSNM) recommendations for maize provided by an ICT-based extension tool called Nutrient Expert (NE) (Pampolino et al., 2012). The NE tool is being developed for extension in the maize belt of Nigeria and ex-ante insights on farmers' preferences for the expected information content and recommendation alternatives from the tool can contribute to optimize its development. We use a choice experiment to provide ex-ante insights on the adoption potentials of site-specific advisory services enabled by digital tools from farmers' perspectives, identify heterogeneous preference classes and the drivers of farmers' preferences.

We contribute to the general literature on agricultural technology adoption, and specifically to the literature on DSTs for improved soil fertility management. Our findings add insights to the R4D literature and are relevant for the development community. The current empirical literature includes ex-post studies that analyze farmers' adoption behavior after technologies have been introduced (e.g. Lambrecht et al., 2014; Mponela et al., 2016; Morello et al., 2018) and a growing number of ex-ante studies that use choice experiments to analyze farmers' adoption behavior in the design stage of a technology (e.g. Lambrecht et al., 2015; Mahadevan and Asafu-Adjaye, 2015; Dalemans et al., 2018; Tarfasa et al., 2018). However, none of the available studies focuses on farmers' adoption of site-specific extension recommendations and also farmers' willingness to accept such recommendations from ICT-based extension tools has not been researched (Fu and Akter, 2016; Verma and Sinha, 2018). The only available empirical study on preferences for ICT-based extension tools focuses on the extension providers rather than the ultimate beneficiaries (farmers) (Kragt and Llewellyn, 2014). Building on Kragt and Llewellyn (2014), we also contribute to the choice experiment literature by extending the application of the methodology in optimizing design of DSTs but with a more rigorous empirical estimation. We specifically take into account both farmers' response error and attribute non-attendance using different econometric models, which is an advancement in comparison with previous choice experiment studies that address only one of these issues (e.g. Kragt, 2013; Coffie et al., 2016; Dalemans et al., 2018; Campbell et al., 2018; Caputo et al., 2018).

The remainder of the paper is organized as follows. In Section 2 we provide some background on maize production, soil fertility and conventional extension in Nigeria as well as the development of a nutrient expert tool. In Section 3 we explain the methodological approach of the paper. In Section 4 we report the results of the empirical analysis and provide a discussion of the results in Section 5. Section 6 concludes the paper.

## 2 Background

### 2.1 Maize production in Nigeria

**Fig. 1:**
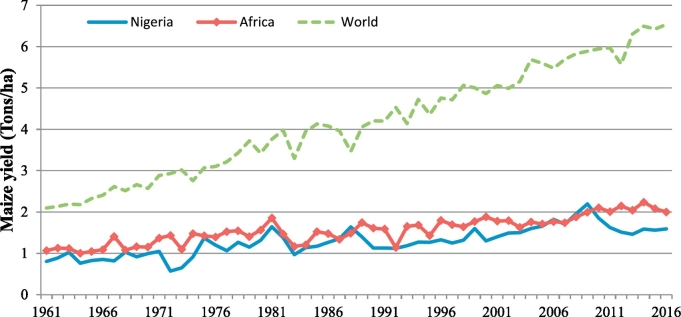
Maize yield trend in Nigeria, Africa and the world at large (FAOSTAT, 2018).

A crop of notable interest for food security and the most widely grown in SSA is maize (van Ittersum et al., 2016). As in other countries in SSA, maize is a very important crop in Nigeria, where it is largely cultivated by smallholder farmers (Abdoulaye et al., 2018). Yet, on-farm yields are low and far below attainable yields in experimental stations, leading to a substantial yield gap (Shehu et al., 2018). Maize yields in Nigeria have consistently lagged behind those in the rest of the world – with maize yield in Nigeria being only one fourth of the average global yield in 2016 – and are currently even lagging behind on the average yield in Africa (Fig. 1).

### 2.2 Soil fertility and conventional extension

The average low maize yield in Nigeria is related to inherent poor soil fertility, and continuous cropping and mining of soil nutrients (Tarfa et al., 2017; Ande et al., 2017). Soil nutrient deficiencies are common with N as the most limiting nutrient for maize production in the Nigerian savannas (Chianu and Tsujii, 2005; Shehu et al., 2018). Fertilizer use to address nutrient deficiencies is low. Average fertilizer use on arable land is estimated to be 8.3 kg nutrient per ha in 2015 (FAOSTAT, 2017). This is despite the commitment of Nigeria and other African countries to increase fertilizer use from 8 to 50 kg nutrients per ha by 2015 (Sanginga and Woomer, 2009; Vanlauwe et al., 2015b). Low fertilizer use has been attributed to market constraints such as a lack of fertilizer availability during the season, high cost of fertilizer, low access to credit, high transportation costs, behavioral constraints such as risk and time preferences, poor knowledge of fertilizer use, as well as to a poor yield responses to fertilizer applications (Chianu and Tsujii, 2005; Sanni and Doppler, 2007; Ande et al., 2017; Tarfa et al., 2017). Although the agricultural extension system is generally weak in Nigeria, considerable extension services are directed to maize production because of its importance for food security (Ande et al., 2017). The extension system provides a general fertilizer recommendation of 120 kg N, 60 kg P_2_O_5_ and 60 kg K_2_O per ha for maize in the Northern guinea savanna of Nigeria (Shehu et al., 2018; Tarfa et al., 2017). Yet, maize farmers use on average only between 40 and 45 kg N per ha (Liverpool-Tasie et al., 2017). The use of this general recommendation is not consistent with the principle of dynamically adjusting fertilizer application based on crop need, and field- and season-specific conditions (Pampolino et al., 2007). In addition, general recommendations may result in fertilizer rates that are sub-optimal from an economic point of view because (expected) marginal returns to fertilizer application are not the same across farmers and fields. Site-specific recommendations may result in fertilizer application rates that allow to better align marginal costs and benefits of fertilizer application, and better account for farmers risk preferences.

### 2.3 Nutrient expert tool

The project ‘Taking Maize Agronomy to Scale (TAMASA)’ is co-developing a user-friendly, scalable nutrient management extension tool, known as Nutrient Expert (NE), with the aim of providing site-specific soil fertility management recommendations to maize farmers in Nigeria, Tanzania and Ethiopia.^1^ This effort is expected to result in a mobile phone-based, easy-to-use and interactive tool that will be used by extension agents to generate fertilizer and management recommendations adapted to the specific situation of an individual farmer's field in real-time (Pampolino et al., 2012). The tool is based on SSNM principles of applying fertilizer according to crop needs by promoting the right fertilizer source (i.e. the type of fertilizer needed), at the right rate (i.e. the amount of fertilizer), at the right time (i.e. the timing of fertilizer application), in the right place (i.e. the availability of nutrients) (4R's of nutrient use). The tool relies on the quantitative evaluation of the fertility of tropical soils (QUEFTS) model to predict the yield responses. The inputs required to generate recommendations include a target maize yield, farmer's current crop management practices (inorganic and organic fertilizer use, variety type, yield etc.), characteristics of the growing environment (water availability, risk of flood/drought etc.), soil characteristics (soil texture, soil color, history of manure use etc.) and prevailing market prices of inputs and maize. A target yield is the attainable yield for a farmer's location estimated by the tool using the information on current crop management practices and characteristics of the growing environment provided by the farmer. However, a farmer has the option of choosing a yield lower than the attainable yield as the target yield. The outputs of the tool include information on SSNM (N, P, K application guide and associated crop management practices) to achieve the target maize yield and a simple profit analysis to compare farmers' current practice and the recommended practices. The tool can take into account the financial situation of farmers by allowing recommendations to be adjusted according to their available budget. The tool development process is expected to consist of data collection (multi-location nutrient omission trials), model development (algorithm, decision rules and programming) and field validation (model testing and refining) (Pampolino and Zingore, 2015). In this paper, we examine farmers' preferences for high-input maize production that is supported by site-specific extension recommendations. This allows to analyze how farmers trade off specific attributes of a high-input, −output, −investment and -risk production system, and generates insights for optimizing the design of the NE tool.

## 3 Methodology

### 3.1 Research area and sampling

The research was conducted in the maize belt of northern Nigeria which covers the northern Guinea, southern Guinea and Sudan savannas, and where agro-ecological conditions are diverse. In this region maize is mainly grown under a smallholder rain-fed cereal-legume cropping system. The predominant cropping system in this area is a cereal-legume system with maize and sorghum as main cereal crops and cowpea, soybean and other legumes often intercropped with cereals and sometimes in rotation. The tillage practice in the system is mostly traditional tillage that involves the use of a hoe and animal traction. Retention of crop residues on fields is not very common because residues are often used as livestock feed and fuel (Manyong et al., 2001; Akinola et al., 2015). The cropping system is characterized by low levels of external input use and low yields. The fertilizer application rate for maize is on average <50 kg N per ha. Yields are on average around 2 tons per ha while the potential maize yield in this area has been estimated to be >5 tons per ha (Manyong et al., 2001; Sanni and Doppler, 2007; Liverpool-Tasie et al., 2017; Abdoulaye et al., 2018). The low-input low-output cropping systems relates to a low yield response to fertilizer and to constraints faced by farmers, including information constraints on optimal input use, high cost of fertilizer, low access to credit, and high transaction costs in acquiring inputs (Manyong, 2001; Chianu and Tsujii, 2005; Sanni and Doppler, 2007; Liverpool-Tasie et al., 2017).

**Fig. 2:**
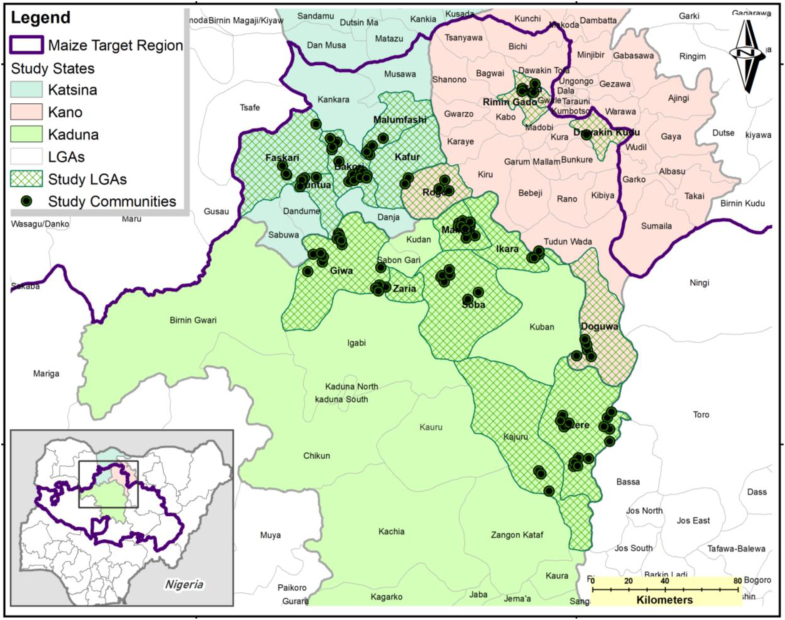
Map of the study area.

For this study, the states of Kaduna, Katsina and Kano (Fig. 2) were purposively selected because of their strategic position in maize production and to pilot research activities for the development of the NE tool. A two-stage sampling design was used to sample households in these states. In the first stage, 22 sampling grids of 10 × 10 km were randomly generated across the primary maize areas in the three states with geospatial inputs to ensure spatial representativeness. These 22 sampled grids include 99 randomly selected villages belonging to 17 local government authorities (LGAs), the administrative unit below the state. All these villages were included in the sample. In the second stage, a sampling frame of maize-producing farm-households was constructed for each of the selected 99 villages. In each of the villages, eight households were randomly selected from a village listing of maize-producing farm-households, which results in a total sample of 792 households. All the selected farm-households are male-headed. Crop production activities in the research area are predominantly carried out by men while women are largely engaged in crop processing activities. Cultural norms such as seclusion of married women among the dominant Hausa ethnic group in most rural communities of the research area is one of the main factors that limit the active participation of women in on-farm activities (Baba and van der Horst, 2018). Also women's poor access to and control over productive resources hinders an active participation and leading role of women in crop production. There is a general believe in the research area that women do not farm (Manyong et al., 2001). The focus on male-headed households limits a detailed consideration of gender issues in this study.

### 3.2 Design and implementation of a choice experiment

In this research area, we implemented a discrete choice experiment (CE) with the 792 sampled farmers during the maize harvest period of 2016 and complemented the CE data with a farm-household survey. A discrete CE is a survey-based method for eliciting preferences of respondents. These preferences are derived from respondents' repeated choices between two or more discrete alternatives of a ‘good’, ‘service’ or ‘course of action’ described by various levels of specific attributes of these products (Pouta et al., 2014). This approach makes it possible to evaluate farmers' preferences for high-input agriculture supported by SSNM recommendations prior to the development of the NE tool and take into account these preferences in designing, fine-tuning and delivering the tool. CEs first emerged in marketing studies and now cut across several disciplines, including agricultural sciences where CEs are increasingly applied in ex-ante agricultural technology adoption studies (Mahadevan and Asafu-Adjaye, 2015; Lambrecht et al., 2015; Coffie et al., 2016; Kassie et al., 2017; Tarfasa et al., 2018; Dalemans et al., 2018). Theoretically, the CE method is based on Lancaster's economic theory of value (Lancaster, 1966) and random utility theory (McFadden, 1974). Practically, collecting CE data entails the identification of relevant attributes, the identification of levels for each of these attributes, an experimental design into different choice sets, the construction of choice cards with these choice sets, and the implementation of the CE among respondents. We discuss these steps below.

#### 3.2.1 Identification of attributes and attribute levels

To identify relevant attributes or technology traits associated with SSNM, we consulted several scientists within and outside the project team and conducted three focus group discussions with farmers.^2^ Ten relevant attributes were identified namely fertilizer application rate, fertilizer application method, fertilizer application timing, fertilizer sources, fertilizer quality, seed type, planting density, expected yield, yield variability, cost of fertilizer and seed. A clear description of these attributes and the range of possible levels of the attributes to be included in the CE were elicited from review of soil fertility management literature and during the consultations. However, only the six most important attributes, as revealed from a ranking of attributes during the focus group discussion and the consultations with scientists, were included in the CE in order to reduce the complexity of the choice tasks from inclusion of too many attributes and limit the occurrence of random non-deterministic choices by farmers (Beck et al., 2013). The attributes and their levels are summarized in Table 1. The first two attributes directly relate to fertilizer use in the context of SSNM. The first attribute is ‘fertilizer application rate’, defined as the quantity of inorganic fertilizer required to supply the necessary nutrients to achieve a target maize yield on a specific field. This is described by three levels: the farmer's current application rate (not site-specific), a site-specific rate below the current rate, and a site-specific rate above the current rate. The second attribute is ‘fertilizer application method’, which relates to how fertilizer is applied to guarantee optimal uptake of nutrients by maize plants and ensure that desired maize yields are attained. The levels of this attribute are broadcasting and dibbling/spot application.

**Table 1:** Attributes and attribute levels.

Attributes	Attribute levels
Fertilizer application rate	Current rate (not site-specific)
	Site-specific fertilizer rate (SSFR): below current rate
	Site-specific fertilizer rate (SSFR): above current rate
Fertilizer application method (FAM)	Broadcasting, Dibbling
Expected yield	1 to 2, 2 to 3, 3 to 4, 4 to 5, 5 to 6 tons/ha
Yield variability (yield risk)	0 (0 in 5 years), 1 (1 in 5 years), 2 (2 in 5 years), 3 (3 in 5 years), 4 (4 in 5 years)
Seed type (ST)	Traditional variety, Improved variety
Cost of fertilizer and seed (CFS)	35,000, 45,000, 55,000, 65,000, 75,000, 85,000 NGN/ha

Note: 305 NGN (Nigerian Naira) is equivalent to 1 USD at the survey time.

The third and fourth attributes relate to returns in terms of yield and variability in yield associated with using SSNM. The third attribute is ‘expected yield’, expressed as average yearly maize yield expected on a hectare over a production period of 5 years. This attribute is defined by five levels, ranging from 1 to 6 tons/ha, carefully selected within the range of attainable maize yields in the research area. The fourth attribute is ‘yield variability’ or yield risk, i.e. the probability of a bad production year. This attribute is described by five levels expressing the number of production years, ranging from 0 to 4 out of 5, maize yield will be below one ton per hectare.

The fifth attribute ‘seed type’ relates to type of maize seed, a vital complementary input in addition to fertilizer to improve maize yields. Fertilizer recommendations are often combined with recommendations on improved seed in extension services due to interaction effects of fertilizer and improved seeds, especially as promoted in integrated soil fertility management (Vanlauwe et al., 2015b). The levels of this attribute are improved seed variety and traditional seed variety.

The last attribute is a monetary attribute defined as the ‘cost of fertilizer and seed’ in local currency (Nigerian Naira - NGN) per hectare. This represents the fertilizer and seed investment cost associated with adopting a given extension recommendation. This attribute is defined by five levels, ranging from 35,000 to 85,000 NGN (115 to 279 USD) per hectare, that were determined based on a range of actual costs incurred on fertilizer and seed during the 2016 growing season, for which information was obtained through focus group discussions and a pilot survey.

#### 3.2.2 Experimental design and choice cards

Based on the selected attributes and attribute levels, the choice experimental design was implemented in Ngene 1.1.2 software to combine the various attribute levels into different pairs of mutually exclusive hypothetical options of soil fertility management (i.e. choice sets or situations) that will be evaluated by farmers. For the experimental design we use a fractional factorial design to allocate the attribute levels into choice sets; more specifically a Bayesian D-efficient design which minimizes the D-error and improves efficiency of the design. As proposed by Scarpa et al. (2013), and to improve efficiency, we conducted a pilot version of the CE with 30 farmers prior to the actual design. For this pilot CE we used an orthogonal design in which priors are fixed to zero. With the data from this pilot CE, a multinomial logit model was estimated and parameter estimates were used as Bayesian priors (random priors distribution) in generating the ultimate D-efficient design. This design resulted in 12 paired choice sets that were randomly blocked into two blocks of six choice sets such that each farmer can easily evaluate six choice sets. The blocking facilitates the implementation among farmers as it reduces the cognitive burden of evaluating too many choice sets and improves the quality of responses.

**Fig. A1:**
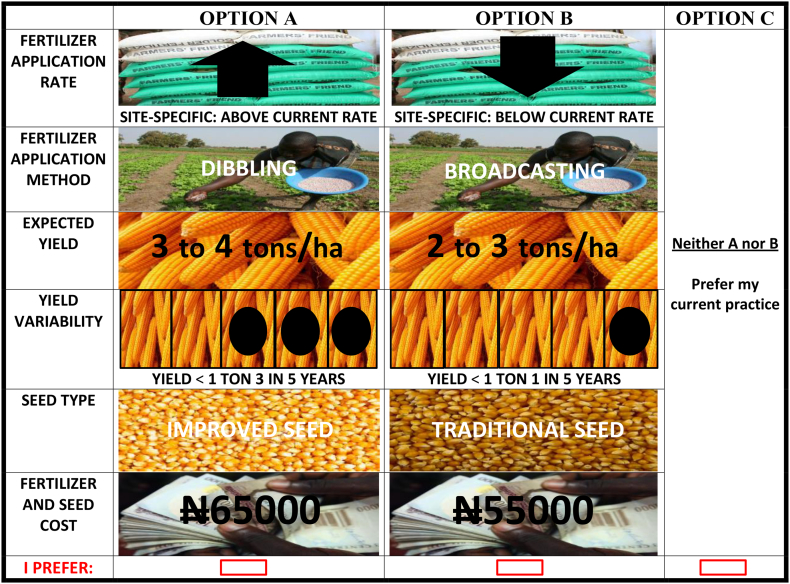
Example of a choice card.

Twelve laminated choice cards were constructed for the 12 paired choice sets – see an example in Fig. A1 in appendix. In order to make the CE more comprehensible among less educated farmers in the sample, we include pictures for different attributes in the choice cards. Each choice card consists of two generic scenarios or alternatives (options A and B) of SSNM recommendations. Each option is defined by six attributes but differs in some attribute levels. A status quo option which represents the current practice of farmers is included in all choice cards as option C. This makes the CE more realistic as farmers have the option of choosing their current practice if it appears superior and reduces potential bias arising from forced choices for options A and B (Lancsar et al., 2017).

#### 3.2.3 CE and survey implementation

In the CE implementation, the experimentally designed hypothetical options of fertilizer recommendations were provided to each farmer in the form of choice cards. Farmers were asked to carefully evaluate the options on each choice card and to choose the most preferred option for each choice card. Each farmer was presented six distinct choice cards and each choice card had three options of fertilizer recommendations (options A, B and C). Within the set of six choice cards presented to each farmer, options A and B vary within and between the cards but option C which represents the farmer's current practice is fixed. The choice of one option (e.g. option B) over the others (options A and C) on a choice card implies that the expected utility of the chosen option exceeds the utility of the other options. Prior to the CE, there was an introductory session in which farmers were sensitized on its purpose, contents and how to correctly participate. As part of the introduction, we used a short cheap talk script (Cummings and Taylor, 1999) to explain to farmers the importance of making truthful choices and thereby limit hypothetical bias arising from divergence between choices made in the hypothetical CE scenarios and (unobserved) actual choices when exposed to site-specific recommendations from ICT-based tools. The text that was used in this cheap talk script is included in Appendix. After the introductory session, the six choice cards were presented one after the other to each farmer by an enumerator and each farmer was specifically asked to carefully examine the three options on each card, and freely make a choice between the three options on each of the six cards. This is on the premise that the technology option that offer the largest expected utility for the farmer will be chosen among the different options available.

The CE was complemented with a farmer survey. The survey questionnaire consists of plot-, household- and community-level components. The modules of the questionnaire include household demographics, access to services, assets, income, fertilizer use, crop production and access to community infrastructure. To improve the quality and timely availability of the data, the survey was implemented using computer-assisted personal interviewing software and tablets.

### 3.3 Econometric framework

The random utility theory behind CEs assumes that the utility of farmer *i* of choosing alternative *j* among all alternatives offered in a choice set *s* is given by an indirect or unobservable utility which consists of deterministic (explainable) and random (unexplainable) components as follows:

(1) $$U_{\mathrm{ijs}}=V_{\mathrm{ijs}}+\varepsilon_{\mathrm{ijs}}=\mathrm{ASC}+\sum_{k=1}^{6}\beta_{i}x_{\mathrm{ijs}}+\varepsilon_{\mathrm{ijs}}$$

Where *U*_*ijs*_ is the *i*^*th*^ farmer's indirect or latent utility, *V*_*ijs*_ is the systematic part of the utility function, *x*_*ijs*_ is a vector of attributes describing alternatives *j* with associated preference parameters *β*_*i*_, *ε*_*ijs*_ is an unobserved random term that is independently and identically distributed (iid) across individuals and alternatives, *ASC* is an alternative-specific constant which represents preferences for the status quo option.

Drawing upon this model, we estimate a latent class model (LCM) with our empirical data. In the context of this study, the LCM assumes that a heterogeneous population of farmers belongs to a discrete number of preference classes, known as latent classes, with each farmer having a positive probability of membership of each class (Kragt and Llewellyn, 2014). The preference parameters in Eq. 1 become class-specific parameters *β*_*c*_. This implies that preferences are homogeneous within each latent class *c* but heterogeneous across classes. Hence, the probability of farmer *i* choosing alternative *j* in choice set *s* is conditional on the farmer's membership of latent class *c*.

(2) $${\mathrm{Pr}}_{\mathrm{ijs}}|c=\frac{\exp\left(\beta_{c}x_{\mathrm{ijs}}\right)}{\sum_{t=1}^{J}\exp\left(\beta_{c}x_{\mathrm{its}}\right)}\;$$

The class membership probability is modeled using a multinomial logit specification as a function of farmer-specific characteristics^3^ known to be relevant for soil fertility-related technology adoption from theory and the empirical literature (Feder et al., 1985; Foster and Rosenzweig, 2010; Chianu and Tsujii, 2005; Lambrecht et al., 2014; Wiredu et al., 2015; Mponela et al., 2016; Morello et al., 2018). The selected variables are age and education level of the farmer, household labor (human capital), membership in a farmer association (social capital), access to off-farm income, access to agricultural credit (financial capital), the value of assets (physical capital), access to extension services and distance to a tarmac road (access to institutions and infrastructure).

(3) $${\mathrm{Pr}}_{\mathrm{ijs}}|c=\frac{\exp\left(\gamma_{c}^{\prime }z_{i}\right)}{\sum_{q=1}^{C}\exp\left(\gamma_{q}^{\prime }z_{i}\right)}\;$$

Where *z*_*i*_ is a vector of farmer-specific characteristics and *γ*_*c*_^′^ is a vector of parameters of *z*_*i*_. Both choice and membership probabilities are jointly estimated with the assumption that scale parameters are normalized to one, as required for identification (Boxall and Adamowicz, 2002).

The ASC is dummy-coded as 1 if a farmer chooses the current practice and 0 otherwise. A negative coefficient for the ASC implies a positive utility of moving away from the current practice to following ICT-enabled SSNM. The categorical attributes are dummy-coded for ease of interpretation of coefficients (Van den Broeck et al., 2017). To improve the explanatory power of the model, we use farmer-specific status quo attribute levels in the estimation (King et al., 2007).

A growing body of literature shows that choice modeling can produce biased estimates of preferences if scale and preference parameters are confounded (Louviere and Eagle, 2006). The implication is that the LCM can yield spurious classes with heterogeneity largely an issue of scale (random choices) and less of taste (preference) (Vermunt and Magidson, 2014). As a robustness check, we estimate a scale-adjusted LCM (SALCM) to address this issue of potential confounding of scale (*λ*_*d*_) and preference (*β*_*c*_) parameters. The choice probability then becomes conditional on an individual farmer's membership of latent preference class *c* and scale class *d*.

(4) $${\mathrm{Pr}}_{\mathrm{ijs}}|c,d=\frac{\exp\left(\lambda_{d}\beta_{c}x_{\mathrm{ijs}}\right)}{\sum_{t=1}^{J}\exp\left(\lambda_{d}\beta_{c}x_{\mathrm{its}}\right)}\;$$

Another source of bias is violation of the continuity axiom of choice. This axiom implies that respondents consider all the attributes of the alternatives in their choice process (Kragt, 2013; Coffie et al., 2016). Violation of this axiom is commonly referred to as attribute non-attendance (ANA) and implies non-compensatory decision making behavior of respondents. In the context of this study, farmers may not make the expected full trade-offs between all attributes of the various alternatives. We rely on self-reported or stated ANA responses of farmers elicited at the end of the CE (Serial-based ANA) and estimate two stated ANA models to check the robustness of our results. The first approach referred to as the conventional ANA model involves constraining parameters of ignored attributes to zero in the utility function, implying that failure to attend to an attribute by a respondent leads to zero marginal utility for that attribute (Kragt, 2013; Campbell et al., 2018; Caputo et al., 2018).

(5) $$U_{\mathrm{ijs}}|c=\mathrm{ASC}+\sum_{k=1}^{6-\tau }\beta_{c}x_{\mathrm{ijs}}+\varepsilon_{\mathrm{ijs}}\;$$

Where *τ* are ignored attributes, as self-reported by farmers. The specialized literature shows that ANA does not necessarily imply zero utility weight for an attribute but often indicates that respondents assign a lower importance to the attribute, and is best captured by a lower magnitude of the marginal utility for non-attenders than attenders (Hess and Hensher, 2010; Kragt, 2013). This motivates the estimation of a second ANA model known as validation ANA model. This model involves estimating two parameters for each attribute depending on whether the attribute is reported to be ignored or considered by respondents in their choice making (Hess and Hensher, 2010; Scarpa et al., 2013; Alemu et al., 2013; Caputo et al., 2018). Following Caputo et al. (2018), the utility coefficients conditional on attendance is indicated with the superscript 1 (*β*_*c*_^1^) and those conditional on non-attendance with superscript 0 (*β*_*c*_^0^).

(6) $$U_{\mathrm{ijs}}|c=\mathrm{ASC}+\sum_{k=1}^{6-\tau }\beta_{c}^{1}x_{\mathrm{ijs}}+\sum_{k=1}^{\tau }\beta_{c}^{0}x_{\mathrm{ijs}}+\varepsilon_{\mathrm{ijs}}\;$$

This approach helps to validate the first ANA model. Based on the validation method, choice behavior of respondents is expected to be in line with their self-reported ignored attributes if the estimated coefficients of ignored attributes are not significantly different from zero.

In summary, we estimate the following models: a standard latent class model (LCM) in STATA 15, a scale-adjusted latent class model (SALCM) in Latent Gold Choice 5.1, a conventional attribute non-attendance model (conventional ANA), and a validation attribute non-attendance model (validation ANA) in NLOGIT 5.

## 4 Results

### 4.1 Descriptive results

Table 2 describes individual-, household- and farm-level characteristics of sampled farmers. Farmers are on average 44.7 years old and have an average of 5.2 years of schooling. Farm-households include on average 1.7 adult men, 1.9 adult women and 5.6 children. Farmers have on average 3.2 ha of land and 19 years of farming experience. About 21% of the sampled farmers have access to credit, 34% are member of a farmer association, 16% produce maize under a contract-farming arrangement and 37% have extension experience from government and or non-government extension service providers. On average farmers apply 127 kg of NPK fertilizer per ha, and 89 kg of urea per ha and 28% of farmers use improved maize seeds, resulting in an average input cost of 39,000 NGN (128 USD) and an average maize yield of 2.1 tons per ha. The application of NPK (15:15:15 and 20:10:10) and urea (46 N) is equivalent to 61 kg N, 19 kg P_2_O_5_ and 19 kg K_2_O per ha, which is below the general recommendation. Farm-households live on average 4.08 km from the nearest tarmac road and the large majority (81%) is located in the northern guinea savanna agro-ecological zone.

**Table 2:** Summary statistics of farmers' characteristics (*N* = 792).

	Description of variable	Mean	SD
Age (years)	Age of household head	44.70	12.03
Education (years)	Years of schooling attained by household head	5.16	6.01
Health of head (%)^a^	Health status of household head	96.43	
Male adults (no.)	Number of male adults in the household	1.70	1.02
Female adults (no.)	Number of female adults in the household	1.87	1.22
Children (no.)	Number of children in the household	5.88	4.49
Credit (%)	Household with access to agricultural credit	20.7	0.40
Member of association (%)	Household belonging to a farmer association	33.71	
Maize contract farming (%)	Household producing maize under contract-farming	16.37	
Extension (%)^b^	Household with access to extension services	37.28	
Farming experience (years)	Years of maize farming	19.11	0.43
Off-farm income (%)	Household with access to off-farm income	94.98	
Farm assets^c^ (1000 NGN)	Value of farm assets	51.36	11.45
Transport assets (1000 NGN)	Value of transport assets	201.85	459.05
Livestock assets (1000 NGN)	Value of livestock assets	394.51	586.67
Durable assets^d^ (1000 NGN)	Value of durable assets	22.66	52.86
Annual income^e^ (1000 NGN)	Household income of the past year	177.63	221.35
Total farm area (ha)	Size of farmland	3.23	3.63
Maize focal plot area^f^ (ha)	Size of maize focal plot	0.82	1.04
Use improved seed (%)	Household cultivating improved maize seed	28.04	
NPK fertilizer (kg/ha)	Quantity of NPK fertilizer applied per hectare	126.96	102.84
Urea fertilizer (kg/ha)	Quantity of urea fertilizer applied per hectare	88.79	95.09
Input cost/ha^g^ (1000 NGN)	Cost of fertilizer and seed	38.61	25.11
Maize-legume intercrop (%)	Maize plot intercropped with legumes	30.15	
Maize yield (tons/ha)	Output of maize per hectare	2.05	0.91
Distance to tarmac road (km)	Distance from homestead to nearest tarmac road	4.08	5.15
Northern guinea savanna (%)	Northern guinea savanna agro-ecological zone	80.71	
Southern guinea savanna (%)	Southern guinea savanna agro-ecological zone	3.40	
Sudan savanna (%)	Sudan savanna agro-ecological zone	15.88	

NGN: 305 NGN (Nigerian Naira) is equivalent to 1 USD at the survey time.

a Percentage of farmers who self-report to be healthy during the past one year.

b Extension experience through a face-to-face contact with extension agents, on-farm trials, field demonstrations or any extension-related training from both government and non-government extension services in the last three years.

c Value of non-land assets, including farm equipment and machinery.

d Value of durable assets such as TV, radio, refrigerator, mobile phone, sewing machine etc.

e Per-adult equivalent household annual income from all sources.

f Maize focal plot is defined as the plot a household considers as their most important maize plot.

g Input cost only refers to cost of fertilizer and seed for maize in the 2016 season.

### 4.2 Econometric results

Before discussing the results in detail, we elaborate on scale heterogeneity and ANA. First, scale heterogeneity is addressed in the SALCM. In this model, the scale factor of scale class one is fixed to unity for identification purposes while that of scale class two is estimated. The latter is very small (0.13), indicating that farmers in scale class two make less consistent choices resulting in higher error variance. As the large majority of farmers (96%) belong to scale class one (and make consistent choices) and the parameter estimate of the scale factor is weakly significant, we can conclude that there is only weak evidence of heterogeneity in scale across the two classes.

Second, the descriptive information in Table 3 shows that 42% of farmers ignored at least one attribute, which justifies the estimation of the ANA model. The results of the validation ANA model show that the choice behavior of farmers in the CE corroborates their self-reported ANA as almost all parameter estimates of the self-reported ignored attributes are not significantly different from zero. This implies that self-reported ANA does not bias the results in the conventional ANA model and that restricting the parameters of ignored attributes to zero works well for our data. This is line with the findings of Caputo et al. (2018) and in contrast to Alemu et al. (2013) on ANA validation models at choice task and serial levels respectively.

**Table 3:** Descriptive information on stated ANA.

# ignored attributes	Share of respondents (%)	Ignored attributes	Share of respondents (%)
0	57.7	Fertilizer application rate	15.1
1	10.4	Fertilizer application method	30.3
2	14.4	Expected yield	4.4
3	16.9	Yield variability	9.1
4	0.7	Seed type	20.4
		Cost of fertilizer and seed	13.1

We estimate four LCMs with two to seven latent classes in order to sufficiently represent the preference heterogeneity in our data. Based on the Akaike Information Criteria (AIC) and the Bayesian Information Criteria (BIC) (Boxall and Adamowicz, 2002), a two-class model is selected as the one with the best fit. The results of the estimated LCMs with two latent classes are presented in Table 4, including the LCM, SALCM, conventional ANA and validation ANA models. The parameter estimates are consistent across the different models, implying robust results. The SALCM has the best fit according to the AIC and BIC but has a weakly identified ASC as indicated by a very large standard error. As this is associated with imprecise estimates (Vermunt and Magidson, 2014),^4^ we base our discussion primarily on the standard LCM which is the second best fit and results in estimates that are comparable with the other models.

**Table 4:** Results of different latent class models estimating farmers' preferences for ICT-based site-specific extension.

	LCM		SALCM		conventional ANA		validation ANA			
							AC	AI	AC	AI
Class	LC1	LC2	LC1	LC2	LC1	LC2	LC1		LC2	
Class probability	64%	36%	65%	35%	63.5%	36.5%	66%		34%	
ASC	−5.667*** (0.703)	−5.263*** (0.609)	−24.105 (31.319)	−9.381 (10.611)	−5.694*** (0.652)	−5.367*** (0.562)	−5.693*** (0.680)		−5.268*** (0.583)	
SSFR (Below current rate)	0.058 (0.077)	0.579*** (0.180)	0.073 (0.079)	0.562*** (0.191)	0.029 (0.082)	0.483*** (0.168)	0.029 (0.078)	0.300* (0.174)	0.499*** (0.186)	0.811** (0.363)
SSFR (Above current rate)	0.246*** (0.076)	−0.156 (0.280)	0.249*** (0.079)	−0.190 (0.291)	0.258*** (0.080)	−0.297 (0.241)	0.295*** (0.079)	0.097 (0.172)	−0.508 (0.399)	0.513 (0.386)
Dibbling	−0.073 (0.057)	−0.351*** (0.126)	−0.085 (0.059)	−0.333** (0.132)	−0.052 (0.065)	−0.398*** (0.133)	−0.068 (0.064)	−0.132 (0.091)	−0.396*** (0.143)	−0.182 (0.209)
Expected yield	0.046** (0.020)	0.243*** (0.071)	0.045** (0.020)	0.270*** (0.074)	0.034* (0.020)	0.233*** (0.048)	0.044** (0.019)	0.071 (0.079)	0.289*** (0.081)	0.169 (0.183)
Yield variability	−0.054** (0.024)	−0.528*** (0.073)	−0.059** (0.025)	−0.542*** (0.077)	−0.046* (0.023)	−0.519*** (0.065)	−0.056** (0.023)	−0.061 (0.058)	−0.561*** (0.088)	−0.629*** (0.130)
Improved seed	0.253*** (0.060)	0.154 (0.147)	0.252*** (0.062)	0.178 (0.157)	0.233*** (0.064)	0.057 (0.141)	0.246*** (0.063)	0.327*** (0.113)	0.093 (0.167)	−0.067 (0.258)
CFS (10000 NGN)	0.029* (0.017)	−0.068* (0.038)	0.028* (0.017)	−0.067* (0.040)	0.038** (0.017)	−0.089*** (0.034)	0.030* (0.016)	−0.041 (0.049)	−0.071 (0.044)	0.195** (0.092)
N	14256		14256		14256		14256			
Log likelihood	−2375.63		−2369.74		−2406.18		−2365.50			
AIC	4803.27		4793.48		4864.40		4811.00			
BIC	4993.46		4912.95		5026.00		5059.70			

LCM = standard latent class model, SALCM = scale-adjusted latent class model; conventional ANA = conventional attribute non-attendance model; validation ANA = validation attribute non-attendance model; LC = latent class; AC = attributes considered or attended to, AI = attributes ignored or non-attended to.

The SALCM model has two scale classes: scale class 1 with a probability of 96% and a scale factor set to unity; scale class 2 with a probability of 4% and a scale factor of 0.13.

Standard error reported between parentheses. Significant coefficients at * *p* < .1, ** *p* < .05 and *** *p* < .01.

The results of the LCM show that the estimated coefficient of the ASC is highly significant and negative for both latent classes of farmers. This implies that overall, farmers have positive preferences for site-specific fertilizer recommendations over the current extension practice. Only in 3% of the choices farmers chose the opt-out, implying they prefer the current practice over the site-specific scenarios of soil fertility management. Both classes have significant positive preferences for site-specific fertilizer application rates. Latent class one farmers (LC1) have a significant positive preference for a site-specific fertilizer rate that is above their current fertilizer application rate, which indicates a preference for moving to a high-input high-output production system. Latent class two farmers (LC2) have a significant positive preference for a site-specific fertilizer rate that is below their current application rate, which indicates a low willingness to move to a high-input high-output production system. The coefficients for seed type show that only LC1 farmers have a positive preference for using an improved seed variety; for LC2 farmers this coefficient is not significant. In addition, in LC1 there is a positive preference for a higher fertilizer and seed cost while in LC2 this is negative. The latter is consistent with the law of a downward sloping input demand curve. The former is not and may seem counterintuitive. This results likely stems from the failure to account for the quality of inputs in the design of the choice experiment, and the intuitive association farmers make between cost and quality of inputs while eliciting their choices during the implementation of the choice experiment. The positive preference for a higher input cost is consistent with a willingness to pay more for higher quality farm inputs. This is in line with Palma et al. (2016) and Lambrecht et al. (2015) who note that a positive cost preference can represent a cue for quality in choice modeling. The coefficient on fertilizer application method (dibbling) is significantly negative in LC2, which indicates these farmers prefer to apply fertilizer through broadcasting rather than through dibbling. The significant positive preference for maize yield and the significant negative preference for yield variability in both classes implies that farmers are interested in site-specific recommendations that result in higher and more stable yields, which is in line with the a priori expectations and with farmers being risk averse.

To gain better insights on the trade-off farmers make between attributes and improve the interpretation of the results, we estimate marginal rates of substitution (MRS) (Greene and Hensher, 2003; Lancsar et al., 2017). With a positive parameter for the cost attribute in LC1, the estimation of MRS in monetary terms is not meaningful for this class. Instead, we estimate MRS in terms of yield variability as a benchmark in order to provide information on the relative importance of attributes. Table 5 shows the estimated MRS which have to be interpreted as the yield risk farmers are willing to accept for an increase in another attribute. The results show that in both classes farmers are willing to accept some yield variability for a higher average yield, but for LC1 farmers this trade-off is on average larger, as revealed from the difference in magnitude of the estimated mean MRS. In addition, LC1 farmers are willing to accept an increased yield risk with the investment in improved seeds and higher fertilizer use stemming from site-specific recommendations, while LC2 farmers are not. The latter farmers are only willing to accept increased yield risk with reduced investment in fertilizer. In summary, LC1 farmers are willing to bear more risk of taking up intensification technologies to improve their maize productivity.

**Table 5:** Marginal rate of substitution (MRS) between yield variability and other attributes for two latent class groups of farmers.

	Expected yield	SSFR (below current rate)	SSFR (above current rate)	Dibbling	Improved seed
LC 1					
Mean	0.860	–	4.572	–	4.693
95% ll	0.056	–	1.093	–	1.572
95% ul	4.179	–	22.673	–	22.108
LC 2					
Mean	0.46	1.097	–	−0.296	–
95% ll	0.238	0.443	–	−1.166	–
95% ul	0.642	1.989	–	0.985	–

MRS is calculated as the negative of the ratio of each attribute coefficient to the yield variability coefficient, ll = lower limit, up = upper limit, 95% confidence intervals are estimated using the Krinsky and Robb method with 2000 draws, MRS is not reported for insignificant coefficients as indicated by ‘-’.

The results of the multinomial logit models estimating the membership in latent classes are reported in Table A2 in the appendix – these results shows that age, education, farmer association, assets, access to agricultural credit, access to extension and distance to road are significantly correlated with class membership. Table 6 shows the differences in individual-, household- and farm-level characteristics between the two classes of farmers defined based on their preferences for ICT-enabled SSNM. We find statistically significant differences in most of the characteristics, which contributes to explaining the differences in preference pattern between the latent classes. The results show that in comparison with LC2, farmers in LC1 are relatively younger, invest more in farm inputs and are generally better-off in terms of income, asset ownership and access to services and institutions such as credit, farmer associations, contract farming arrangements, and extension services. This is in line with a large part of the technology adoption literature pointing to more-endowed farmers being more likely to adopt improved farm technologies and to the importance of association membership and extension services in driving technology adoption (Kuehne et al., 2017; Lambrecht et al., 2014). Farmers in LC2 appear better-off in terms of education and access to roads. Education is often (but not always) associated with a higher likelihood of adopting new technologies – it is not in our case. The benefits of education in enhancing learning processes of a new technology might be minimal for technologies with traits that are familiar to the end-users, which likely applies for fertilizer use. Access to roads is often observed to benefit technology adoption because of reduced transport costs in input purchase but it may have no effect for technologies that are less input intensive. In terms of farming experience, there are no significant differences between the two classes of farmers. Given the observed differences, we can describe LC1 farmers as more resource endowed farmers and LC2 farmers as less resource endowed, and further explain the observed preference patterns.

**Table 6:** Farmer characteristics by preference classes.

	Latent class 1 (*N* = 507)		Latent class 2 (*N* = 285)		
	Mean	SD	Mean	SD	Sig.
Age of head	43.52	11.64	46.90	12.41	***
Education of head	4.37	5.68	6.63	6.30	***
Health of head	96.51		96.30		
Male adults	1.70	1.15	1.68	0.71	
Female adults	1.89	1.31	1.81	1.04	
Children	6.02	4.72	5.62	3.99	***
Access to credit	26.68		9.72		***
Member of association	40.40		21.30		***
Maize contract farming	17.96		13.43		***
Farming experience	19.12	10.48	19.10	10.68	
Extension experience	39.65		32.87		***
Access to off-farm income	96.51		92.13		***
Farm assets	60.68	132.35	34.40	67.70	***
Transport assets	227.01	489.86	158.01	394.69	***
Livestock assets	439.94	651.94	292.57	382.21	***
Durable assets	24.41	63.65	19.41	20.51	***
Annual income	192.72	244.84	149.62	165.07	***
Total farm area	3.19	3.48	3.32	3.86	*
Maize focal plot area	0.80	1.04	0.84	1.03	**
Use improved maize	30.92		22.69		***
NPK fertilizer	125.4	101.83	129.85	104.41	**
Urea fertilizer	94.59	94.42	78.01	95.18	***
Input cost/ha	39.51	25.64	36.93	23.94	***
Maize-legume intercrop	28.93		32.41		***
Yield	2.1	0.92	2.0	0.90	***
Distance to tarmac road	4.78	5.95	2.81	2.71	***
Northern guinea savanna	81.55		79.17		***
Southern guinea savanna	3.24		3.70		
Sudan savanna	15.21		17.13		***

* p < .1, ** p < .05, ^⁎⁎⁎^ p < .01 independent sample *t*-tests of significant differences between the two classes of farmers, Variables are as described in Table 2.

## 5 Discussion

We find that farmers are in general favorably disposed to site-specific extension. This suggests that farmers recognize that their production conditions are heterogeneous and that they are open to soil fertility management recommendations that are tailored to their specific growing conditions and derived from DSTs (Rose et al., 2016). However, farmers have heterogeneous preferences for SSNM recommendations and this observed heterogeneity is correlated with farmers' resource endowments and access to services. We identify two groups of farmers (latent classes) with different preferences. The first group (LC1 representing 64% of the sample) includes innovators or strong potential adopters of SSNM recommendations. Farmers in this group are generally better-off, less sensitive to risk, are more willing to invest in a high-input maize production system, and have no aversion for a more labor-intensive production technique with higher expected returns. This is in line with the expectation that better-off farmers are more responsive to new technologies despite the riskier outcomes of new technologies (Foster and Rosenzweig, 2010). The second group (LC 2 representing 36% of the sample) includes more conservative farmers or weak potential adopters. Farmers in this group have lower incomes and lower productive assets, are more sensitive to yield variability, and prefer less capital and labor-intensive production techniques.

Both the strong and weak potential adopters exhibit strong positive preferences for higher yield, which is consistent with other CE studies that reveal maize farmers' preferences for high yielding technologies (Ortega et al., 2016; Kassie et al., 2017). In addition, they both exhibit disutility for risk, which signals a safety-first behavior to smooth income and consumption (Feder et al., 1985). Yet, the weak potential adopters are less willing (or able) to accept increased yield risk for an increase in yield level (or more willing to forego yield gains for stability in yield) than the strong potential adopters. This is likely related to the observation that weak potential adopters have less resources such as income and assets, and a lower access to credit and extension. They are therefore less likely to accept riskier recommendations compared to the strong potential adopters. This implies that the adoption behavior of farmers and their fertilizer investment decisions are not only influenced by expected profits, which is determined by an increased input cost and an expected yield response to fertilizer, but also by the expected risk exposure associated with high-input high-output production systems. This is in line with the finding of Coffie et al. (2016) that risk exposure negatively affects farmers' preferences for agronomic practices.

The weak potential adopters are averse to labor-intensive fertilizer application methods and to higher yielding intensification options with high cost implications. This is in line with the findings of Coffie et al. (2016) and reaffirms the issue of labor constraints for agricultural technology adoption. The strong potential adopters prefer high yielding intensification options with high investment costs, which indicates their willingness to invest in high-input high-output production systems. These findings imply that less endowed and more risk averse farmers are better served with recommendations that do not involve large expenditures and avoid large yield fluctuations, while better endowed and less risk averse farmers are more likely to follow extension advice with a high-input high-output logic.

From a methodological point of view, we show that it is worthwhile to ensure robustness of results by addressing issues of heterogeneity in error variances and ANA in CE studies. As differences in scale imply differences in choice consistency (Lancsar et al., 2017), this should motivate studies to take into account scale heterogeneity to avoid biased estimates of preferences and spurious preference classes (Thiene et al., 2012; Dalemans et al., 2018). We find that the majority of farmers exhibit consistent choices, which is not surprising as they are largely familiar with the attribute and attribute levels presented in the CE and can readily express their preferences. This is in line with Czajkowski et al. (2015) who note that respondents have a more deterministic choice process from an appreciable level of information and experience on the attributes of a product being valued. Failure to account for ANA is an additional possible source of bias in discrete CEs (Kragt, 2013; Coffie et al., 2016; Hess and Hensher, 2010; Caputo et al., 2018). The estimation of an ANA model validates our finding on the preference for higher yielding recommendations with higher investment costs for the strong potential adopters. Such a result could also stem from non-attendance to the cost attribute (as in Campbell et al., 2018) but this is ruled out in the ANA model. Overall, our results are consistent across all the models, which suggests that any possible bias from scale and ANA issues is relatively small. However, this may not always be the case for other studies that do not account for these issues.

Finally, our results entail some specific implications for the development of the NE and similar tools as well as broader policy implications. The direct implication of the farmers' homogenous preferences for high yielding recommendations and risk aversion for the design of ICT-based extension tools is that in the development process, more attention should be paid on ensuring that tools are robust in estimating expected yields for farmers. Most importantly, our results strongly indicate the need to optimize the design of tools to allow for a feature/module for providing information on yield variability (riskiness of expected outcomes) and not only on attainable yield levels to help farmers make better informed decisions. This is rarely taken into account as most DSTs are designed to produce recommendations for farmers on the basis of an expected yield level without providing further information on the uncertainty of the expected outcomes. Therefore, improving the design of extension tools to enable the provision of information on the riskiness of expected yields will be more rewarding for farmers. This is especially the case for farmers who are more risk averse, are less resource-endowed, are not associated in farmer groups, and have no access to credit and other services. In addition, our results point to the need for extension services that are designed to take into account the heterogeneity in farmers' behavioral responses (Lopez-Ridaura et al., 2018). This implies flexibility in extension tools to switch between low-investment low-risk recommendations, and high-investment high-risk recommendations, depending on the risk and investment profile of the individual farmer. In terms of broader policy implications, farmers' general interest in site-specific recommendations from ICT-based tools lends credence to the theoretical motivation for addressing information inefficiencies in agriculture using digital technologies (Janssen et al., 2017; Verma and Sinha, 2018). Digital inclusion policies to bridge the digital divide can include fostering the use of digital technologies in providing quality extension to farmers. The use of ICT-based extension tools that are farm- and field-specific and flexibly take into account farmers' needs may integrate complementary services – such as credit provision, subsidized inputs and insurance schemes – that are well-targeted and increase the uptake of extension recommendations by farmers as well the efficiency of service provision to farmers.

## 6 Conclusion

In this paper, we analyze farmers' preferences for high-input maize production supported by site-specific nutrient management recommendations provided by ICT-based extension tools such as Nutrient Expert that is being developed for extension services in the maize belt of Nigeria. We use a discrete choice experiment to provide ex-ante insights on the adoption potential of ICT-based site-specific extension services on soil fertility management from the perspective of farmers and with the aim to inform the design of DSTs. The choice experiment was carried out, along with a farmer survey, among 792 farmers in three states in the maize belt of Nigeria. Different econometric models are used to control for attribute non-attendance and account for class as well as scale heterogeneity in preferences. The findings reveal that farmers have strong preferences to switch from general to ICT-enabled site-specific soil fertility management recommendations. We find substantial heterogeneity in farmer preferences for extension recommendations and distinguish between strong and weak potential adopters of more intensified maize production. Strong potential adopters are better-off farmers with higher incomes, more assets and better access to services; they are less sensitive to risk and have higher preferences for investing in farm inputs and more capital- and labor-intensive production systems with higher expected return, even at a higher risk in terms of yield variability. Weak potential adopters are more conservative farmers with lower incomes and less productive assets; they are more sensitive to yield variability, and prefer less capital- and labor-intensive production techniques with a lower but more stable return. In general, our findings imply that farmers in the research area support the use of ICT-based site-specific extension services, which calls for agricultural extension programs to contribute to closing the digital divide through the inclusion of ICT-based technologies in the extension system. More specifically, our findings document the importance of flexible extension systems that take into account the willingness and ability of farmers to invest in high-input production systems and to take risk, and correctly inform farmers on expected yield and returns as well as on the variability in yield and potential losses.

## Figures and Tables

**Table A1:** Latent class model of farmers' preferences for ICT-based site-specific extension (without membership function)^a^.

	LCM		SALCM		conventional ANA		validation ANA			
							AC	AI	AC	AI
Class	LC1	LC2	LC1	LC2	LC1	LC2	LC1		LC2	
Class probability	63%	37%	80%	20%	64%	36%	61%		39%	
ASC^b^	−4.748*** (0.388)	−20.051 (393.955)	−60.379 (48.411)	−124.727 (180.966)	−4.782*** (0.387)	−35.647 (0.2D + 07)	−5.502*** (0.747)		−5.501*** (0.669)	
SSFR (Below current rate)	0.125 (0.084)	0.328* (0.177)	0.445*** (0.145)	−0.434 (0.539)	0.111 (0.090)	0.227 (0.200)	0.065 (0.090)	0.258 (0.192)	0.304* (0.171)	0.846** (0.342)
SSFR (Above current rate)	0.270*** (0.082)	−0.229 (0.297)	0.426** (0.179)	−0.531 (0.719)	0.271*** (0.084)	−0.357 (0.281)	0.339*** (0.090)	0.056 (0.203)	−0.467 (0.364)	0.545 (0.417)
Dibbling	−0.081 (0.061)	−0.303** (0.129)	−0.402*** (0.129)	−0.978 (1.187)	−0.074 (0.071)	−0.355** (0.148)	−0.080 (0.071)	−0.125 (0.101)	−0.294** (0.138)	−0.149 (0.192)
Expected yield	0.047** (0.022)	0.220*** (0.063)	0.147*** (0.037)	1.476* (0.814)	0.040* (0.022)	0.216*** (0.051)	0.037* (0.022)	0.0674 (0.095)	0.250*** (0.072)	0.162 (0.186)
Yield variability	−0.047* (0.026)	−0.512*** (0.086)	−0.478*** (0.098)	0.121 (0.546)	−0.040 (0.028)	−0.532*** (0.093)	−0.036 (0.029)	−0.075 (0.067)	−0.512*** (0.090)	−0.522*** (0.127)
Improved seed	0.279*** (0.063)	0.031 (0.154)	0.116 (0.122)	5.534** (2.563)	0.273*** (0.067)	−0.080 (0.184)	0.290*** (0.072)	0.317** (0.123)	−0.023 (0.156)	−0.001 (0.224)
CFS (10,000 NGN)	0.032* (0.017)	−0.070* (0.040)	−0.052 (0.036)	0.207 (0.129)	0.037** (0.019)	−0.089** (0.038)	0.034* (0.019)	−0.051 (0.055)	−0.063 (0.039)	0.182** (0.090)
Log likelihood	−2405.50		−2391.00		−4067.06		−4067.06			
AIC	4845.00		4820.00		4895.20		4856.90			
BIC	4969.36		4904.07		5000.90		5049.60			

LCM = standard latent class model, SALCM = scale-adjusted latent class model; conventional ANA = conventional attribute non-attendance model; validation ANA = validation attribute non-attendance model; LC = latent class; AC = attributes considered or attended to, AI = attributes ignored or non-attended to.

Number of observations is 14,256.

SALCM has two scale classes. Scale class 1 has class probability of 48% and a scale factor set to unity. Scale class 2 has class probability of 52% and a scale factor of 0.08.

Standard error reported between parentheses.

Significant coefficients at * *p* < .1, ** *p* < .05 and *** *p* < .01.

a Without membership function, the signs and significance of coefficients as well as latent classes closely compares to the results with membership function except for SALCM.

b ASC is weakly identified in SALCM and class 2 of the other models as can be seen from the large values of the estimates due to a non-convergence challenge.

**Table A2:** Results of multinomial logit models estimating membership function.

	LCM	SALCM	conventional ANA	validation ANA
Constant	−2.953* (1.587)	−1.526* (0.813)	−2.818* (1.511)	−2.214 (1.440)
Age	−0.046*** (0.015)	−0.024*** (0.008)	−0.043*** (0.014)	−0.049*** (0.014)
Education	−0.089*** (0.030)	−0.046*** (0.016)	−0.079*** (0.026)	−0.088*** (0.026)
Labor	0.105 (0.102)	0.066 (0.052)	0.093 (0.110)	0.108 (0.093)
Farmer association	0.747** (0.372)	0.410** (0.193)	0.776** (0.336)	0.794** (0.336)
Off-farm income	0.699 (0.596)	0.345 (0.306)	0.539 (0.591)	0.565 (0.563)
Assets	0.318*** (0.130)	0.181*** (0.066)	0.312*** (0.119)	0.279** (0.113)
Agricultural credit	1.175*** (0.452)	0.620*** (0.229)	1.068** (0.423)	1.188*** (0.432)
Extension	0.671** (0.315)	0.331** (0.162)	0.460 (0.296)	0.729** (0.300)
Distance to road	0.132*** (0.049)	0.060*** (0.023)	0.112*** (0.043)	0.124*** (0.044)

Significant coefficients at * p < .1, ** p < .05 and *** p < .01, Latent class 2 as reference class.

## Appendix A Appendix

### A.1 Script for implementation of choice experiment

Dear farmer,

In a bid to serve you with SITE-SPECIFIC extension services in which recommendations are tailored to your field-specific conditions instead of the conventional GENERAL extension recommendations, your inputs are highly needed to optimize the design of an ICT-enabled extension tool “Nutrient Expert” that is being developed. This tool will enable extension service providers easily deliver site-specific nutrient management (SSNM) recommendations to you which will help you make better informed decisions on soil fertility and crop management. I will guide you through an exercise in which you will have the opportunity to choose soil fertility management options for your maize production. These options are defined using six attributes namely fertilizer application rate, fertilizer application method, expected yield, yield variability, seed type and cost of fertilizer and seed. (At this point, kindly show the farmer a card containing the six attributes and attribute levels and also, sample of a choice card with explanations to ensure that the farmer fully understands the choice experiment). I will now offer you six distinct choice cards one after the other and each choice card contains two hypothetical scenarios of SSNM (options A and B) and your current practice (option C). The aim is for you to choose one option that you prefer from the three options on each card and this will require you to objectively reflect on the attribute levels of the two hypothetical scenarios of SSNM in comparison with your current practice. You are to carefully go through and evaluate the options on each card that I will present to you and indicate the option you prefer between the three options on each of the cards. Even though this exercise entails hypothetical options of soil fertility management, kindly make very truthful choices as if these were real choices that have real cost implications. This is to ensure that the choices an individual makes in this hypothetical exercise are not different from the actual choices if such individual were exposed to real site-specific recommendations from the extension tool being developed.

## Footnotes

1 The development of the NE tool is a collaborative effort of International Plant Nutrition Institute (IPNI), International Maize and Wheat Improvement Centre (CIMMYT), International Institute of Tropical Agriculture (IITA), extension service providers, national institutes, government agencies, input dealers and farmers with IPNI leading the process.

2 This includes personal communication with a scientist from IPNI who is leading the tool development activities in Nigeria and four other scientists who are working in the research area. During focus group discussions, farmers were asked about fertilizer use in general (e.g. questions on whether they use fertilizer, at what rate, how often, with what method, awareness/use of fertilizer recommendations, yield with and without fertilizer, fertilizer use constraints etc.) and the range of attributes they consider relevant for soil fertility management

3 Some authors advocate the estimation of LCMs without a class membership function (Van den Broeck et al., 2017; Dalemans et al., 2018). With our data, this results in convergence problems and less intuitive results – the results of models without a membership functions are shown in Table A1 in appendix but are not discussed in the text.

4 The issue of weak identification is common in LCM and often results from model estimation algorithm converging on local maxima instead of global maximum. As recommended and implemented in other empirical studies that used SALCM (Vermunt and Magidson, 2014; Thiene et al., 2012), we tried various values of starting sets and iterations per set to achieve convergence on global maximum but ASC was still weakly identified.
